# Training in Basic Life Support and Bystander-Performed Cardiopulmonary Resuscitation and Survival in Out-of-Hospital Cardiac Arrests in Denmark, 2005 to 2019

**DOI:** 10.1001/jamanetworkopen.2023.3338

**Published:** 2023-03-16

**Authors:** Theo Walther Jensen, Annette Kjær Ersbøll, Fredrik Folke, Signe Amalie Wolthers, Mikkel Porsborg Andersen, Stig Nikolaj Blomberg, Lars Bredevang Andersen, Freddy Lippert, Christian Torp-Pedersen, Helle Collatz Christensen

**Affiliations:** 1 Prehospital Center Region Zealand, Næstved, Denmark; 2 Department of Clinical Medicine, University of Copenhagen, Copenhagen, Denmark; 3 Copenhagen Emergency Medical Services, Copenhagen, Denmark; 4 National Institute of Public Health, University of Southern Denmark, Copenhagen, Denmark; 5 Department of Cardiology, Herlev Gentofte University Hospital, Gentofte, Denmark; 6 Department of Cardiology, Nordsjaellands Hospital, Hillerød, Denmark; 7 Department of Clinical Medicine, Aalborg University Hospital, University of Copenhagen, Copenhagen, Denmark; 8 Danish Clinical Quality Program (RKKP), National Clinical Registries, Department of Clinical Medicine, Denmark

## Abstract

**Importance:**

Strategies to improve survival from out-of-hospital cardiac arrest (OHCA) include mass education of laypersons with no official duty to respond to OHCA. In Denmark, basic life support (BLS) course attendance has been mandated by law in October 2006 for obtaining a driver’s license for all vehicles and in vocational education programs.

**Objectives:**

To examine the association between yearly BLS course participation rate and bystander cardiopulmonary resuscitation (CPR) and 30-day survival from OHCA and to examine if bystander CPR rate acted as a mediator on the association between mass education of laypersons in BLS and survival from OHCA.

**Design, Setting, and Participants:**

This cohort study included outcomes for all OHCA incidents from the Danish Cardiac Arrest Register between 2005 and 2019. Data concerning BLS course participation were supplied by the major Danish BLS course providers.

**Main Outcomes and Measures:**

The main outcome was 30-day survival of patients who experienced OHCA. Logistic regression analysis was used to examine the association between BLS training rate, bystander CPR rate, and survival, and a bayesian mediation analysis was conducted to examine mediation.

**Results:**

A total of 51 057 OHCA incidents and 2 717 933 course certificates were included. The study showed that the annual 30-day survival from OHCA increased by 14% (odds ratio [OR], 1.14; 95% CI, 1.10-1.18; *P* < .001) when BLS course participation rate increased by 5% in analysis adjusted for initial rhythm, automatic external defibrillator use, and mean age. An average mediated proportion of 0.39 (95% QBCI, 0.049-0.818; *P* = .01). In other words, the last result indicated that 39% of the association between mass educating laypersons in BLS and survival was mediated through an increased bystander CPR rate.

**Conclusions and Relevance:**

In this cohort study of Danish BLS course participation and survival, a positive association was found between annual rate of mass education in BLS and 30-day survival from OHCA. The association of BLS course participation rate on 30-day survival was mediated by the bystander CPR rate; approximately 60% of the association of BLS course participation rate on 30-day survival was based on factors other than increased CPR rates.

## Key Points

**Question:**

Is mass education of laypersons in basic life support (BLS) associated with survival from out-of-hospital cardiac arrest (OHCA) 10 years after implementation of a law mandating BLS training for recipients of a driver’s license in Denmark?

**Findings:**

In this cohort study including 51 057 OHCA events, there was a positive association between annual BLS course participation rate and 30-day survival from OHCA in the general population. The association of BLS course participation and survival was mediated in part by an increased rate of bystander cardiopulmonary resuscitation.

**Meaning:**

These results suggest that laws mandating BLS course attendance for driver’s license registration were associated with improved survival and may work partly through increased cardiopulmonary resuscitation rates.

## Teaser

This cohort study of administrative and health records in Denmark examines the association of legally mandated cardiopulmonary resuscitation (CPR) courses with out-of-hospital cardiac arrest survival and assesses the potential mediating role of bystander CPR rate.

## Introduction

Out-of-hospital cardiac arrest (OHCA) is both a cause of many individual tragedies and a socioeconomic burden. Survival from OHCA to discharge is approximately 8% in most of Europe.^1^ Over the past decades several international organizations have developed strategies to improve survival from OHCA, which include mass education of laypersons with no official duty to respond to OHCA in basic life support (BLS).^2^ Efforts to increase survival have long followed so-called “chain of survival” guidelines that lay out components or actions known to increase survival from OHCA.^1,3^ OHCAs in Denmark are registered and reported in the Danish Cardiac Arrest Register (DCAR).^4^ Since the first registration in 2001 in the DCAR, an increase in 30-day survival has been seen, from approximately 4% of cases in 2001 to 14% in 2020.^4,5^ The increase in survival has been linked to decreasing response times of the emergency medical services (EMS) and national initiatives targeting both EMS dispatch centers and laypersons with no official duty to respond to OHCA.^6,7^

In Denmark, several initiatives to increase the quantity of individuals trained in BLS and automatic external defibrillator (AED) application have been implemented on a national level. The initiatives include a fully implemented public access AED program linked with the EMS dispatch system initiated in 2004, legally mandated BLS course attendance in several national educational institutions initiated in 2006, introduction of a national EMS dispatch telephone-assisted cardiopulmonary resuscitation (CPR) protocol staffed by health care professionals in 2011, and a national mobile phone application alerting bystanders in the relative proximity of an OHCA incident initiated in 2019.^8,9,10,11,12,13,14^ The initiation of mandated BLS course attendance in October 2006 for obtaining a driver’s license for all vehicles was the start of a national mass education campaign.^8,9^ The total of BLS course participation certificates earned drastically increased during the implementation period from 2006 to 2009, after which course participation rates stabilized around 3% to 4% of the adult Danish population annually.^9^ In 2019, 10 years after the initiation of mandated BLS courses, approximately 40% of the Danish population have been trained, and BLS course attendance has become a normal part of regular civil leisure activities in Denmark.^9,15^ BLS courses in Denmark are based on European Resuscitation Council (ERC) guidelines for resuscitation and lasted a minimum of four hours. The ERC has established standardized requirements for BLS courses and what participants are expected to learn during a BLS course.^16,17,18,19,20,21^ ERC guidelines include 7 steps or actions recommended for bystanders witnessing an OHCA, including safety (of the bystander), response (bystanders check for response), airway (bystander creates patent airways), breathing (bystander look, listen, and feel for breath), alerting the EMS of OHCA (if no response and absence of normal breathing), send for AED (if more than 1 bystander is present), and circulation (bystander initiates CPR, 30 compressions and 2 mouth-to-mouth ventilations).^21^ Mass education of laypersons in BLS combined with integrating laypersons and the EMS system, is known to positively affects bystander CPR rates and to some extent patient survival.^22,23^ BLS for laypersons is targeting the first 3 links in the chain of survival—early recognition of OHCA and alerting EMS, bystander-initiated CPR, and use of AEDs.^1,22^ Measuring how mass education may affect outcomes for OHCA is challenging as there is obviously no physiological mechanism explaining the connection between teaching all the components of BLS and survival from OHCA.

A scoping review conducted in 2021^22^ and a meta-analysis in 2020^23^ advocate for using changes in bystander CPR rate as the appropriate measure for evaluating BLS mass education programs. This suggests that an association between BLS mass education and survival is mainly mediated through an increased bystander CPR. The correlation between BLS mass education on bystander CPR and 30-day survival have not been quantified,^1^ and BLS covers other elements than increasing CPR rates. There is evidence that BLS courses also have increased early recognition, early call for EMS, higher CPR quality, improved communication with the EMS dispatch center, and increased AED usage, resulting in higher rate of shockable rhythm at EMS arrival.^24^ It is further argued that a community where BLS training is common there might be a consequence of increased social awareness of recognition and acting, even among those whom have not participated in BLS training.^24^ Although these elements are challenging to quantify, it is likely that some of the correlation between mass education in BLS is not directly linked to increased bystander CPR rate.

The aim of this study was to examine the association between yearly BLS course participation rate, bystander CPR, and 30-day survival from OHCA at a national level, following more than 10 years of legally mandated BLS course mass education programs. The secondary aim was to quantify mediation by bystander CPR between the association of mass education of laypersons in BLS and survival from OHCA.

## Methods

This nationwide, register-based cohort study comprised all OHCAs in Denmark between January 1, 2005, and December 31, 2019. Data are presented on an annually aggregated national level. Danish regulation requires no ethical approval when analyzing and handling aggregated data from official registers from Statistics Denmark. The storage of BLS course data was approved by the responsible institute of the Capital Region of Denmark in accordance with the General Data Protection Regulation. This study followed the Strengthening the Reporting of Observational Studies in Epidemiology (STROBE) reporting guideline.

### Setting

Denmark has a land area of 42 933 km^2^ and a population of approximately 5.9 million people as of 2022. Health care is provided free of charge and is paid through direct and indirect taxation. Danish citizens are assigned a specific personal 10-digit Central Person Register Number (CPR-Number), which contains the age and sex of each registered person.^25^

### Data Collection

#### OHCA Data

Verified OHCAs were collected from the DCAR.^5^ For each OHCA in DCAR, information was obtained on bystander-initiated CPR, 30-day survival, whether the OHCA was witnessed by EMS or bystanders, whether the victim was defibrillated by an AED (by non-EMS), and initial rhythm.

#### BLS Education Data

Data concerning BLS education were collected in the period between January 1, 2016, to December 31, 2019, from the national First Aid Council of Denmark and the Danish Heart Foundation Give Life campaign as described in a previous study.^8^ BLS certificates were collected with the social security number from 2016 to 2019 and the number of individual persons who had attained a certificate was verified. From 2005 to 2016, the only data source regarding numbers of BLS participants was the total number of issued certificates collected. This data could be verified by CPR-Number to identify the correct number of corresponding individuals.

#### Extrapolating Years Prior to Verifications of Individuals

There were 4 years, 2016 through 2019, of verified totals of individuals and corresponding number of certificates. To include the years prior to 2016, an extrapolation was conducted to estimate the number of individuals based in the number of issued certificates. Due to the nature of available data a linear ratio extrapolation method^26^ was used. It was assumed that the previous years had the same overall number of new individuals entering compared with the number of individuals receiving recertification training. The extrapolation was calculated by dividing the number of issued certificates from 2005 to 2016 by a factor based on average ratio of individuals to course certificates from 2016 to 2019, equaling a proportion of 0.74. To test the robustness of conclusions using the extrapolation, a sensitivity analysis was conducted on the final model.

#### Exposure, Outcomes, and Confounders

Three models were presented to understand the association between the proportion of BLS trained individuals and 30-day survival from OHCA and bystander CPR rate. The models and included risk factors were based on a relevant 2020 meta-analysis.^27^ The considered risk factors were initial rhythm at OHCA, location of OHCA (public or domestic), non-EMS AED application, witnessed status, EMS response time, average age of OHCA victims, shockable rhythm (proportion of all OHCAs), and the introduction of telephone-assisted CPR protocol.

**Figure 1.**
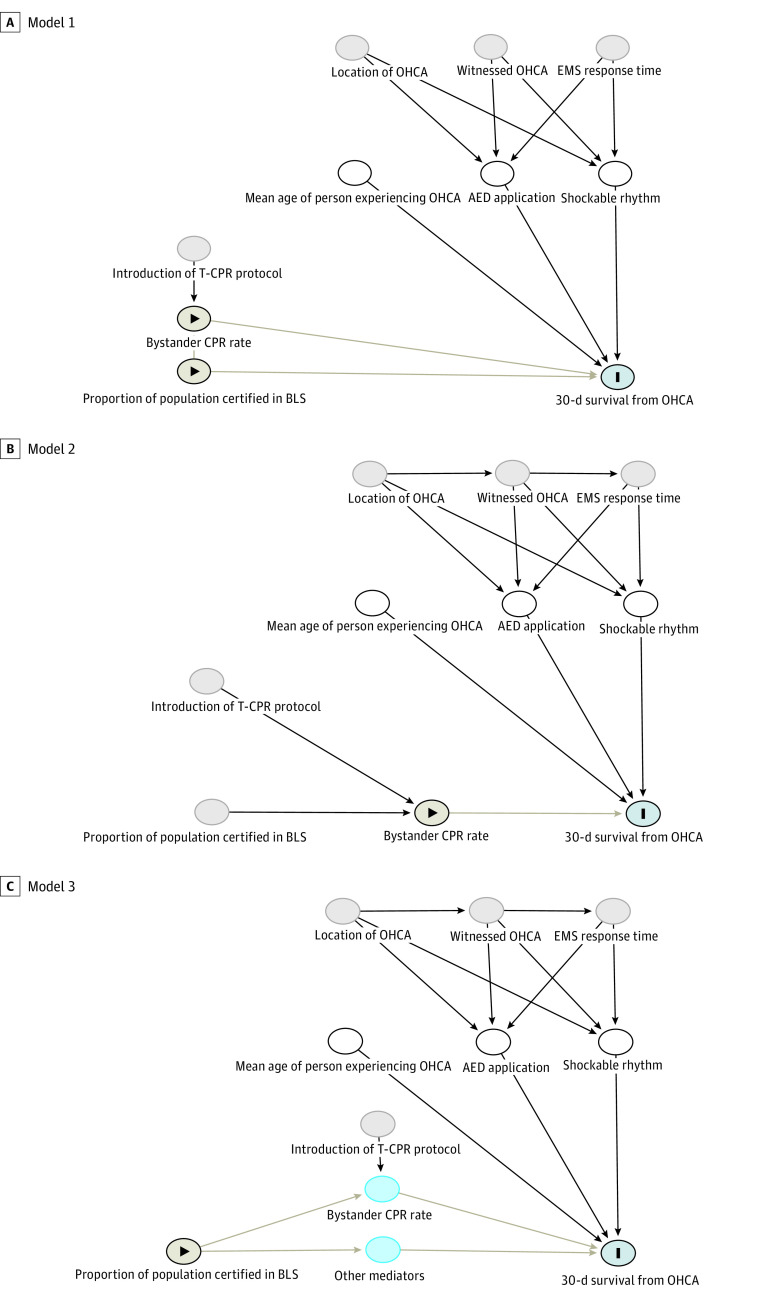
Directed Acyclic Graphs Model Visualizations AED indicates automatic external defibrillator; BLS, basic life support; EMS, emergency medical services; OHCA, out-of-hospital cardiac arrest; T-CPR, cardiopulmonary resuscitation. Pale lines represent relationships between main independent and dependent variables.

After examining these variables together, models were adjusted for average age of OHCA victims, initial rhythm at OHCA, and AED application. Directed acyclic graphs were used to illustrate correlations between included variables (Figure 1). Model 1 included both BLS participation rates and bystander CPR rate as exposure variables. The measure for BLS participation rates was the proportion of the Danish population attending BLS courses annually (on a scale of 0-1). The measure for bystander CPR rate was the proportion of OHCAs where non-EMS persons provided CPR (0-1 scale). Model 2 included only bystander CPR rate as an explanatory variable, acting as proxy for BLS participation rates. Model 3 included only BLS participation rates as an explanatory variable.

### Statistical Analysis

The outcome variables were annual rates of 30-day survival from OHCA and bystander-initiated CPR. Results are presented with odds ratios (OR) and the corresponding 95% CIs. Logistic regression was conducted using the generalized linear model function with a logit link in R statistical software version 4.0.5 (R Project for Statistical Computing). Sensitivity analysis was conducted on a model including all parameters using the Sensemark sensitivity analysis program in R statistical software, which provides a robustness value.^28^ A bayesian mediation analysis was conducted to test if bystander CPR was a mediator in the association between increasing levels of BLS participation rate and 30-day survival rate. The mediator analysis was performed with the statistical package Mediator in R.^29^ A measure of significance is presented together with parameter estimates and 95% quasi-bayesian approximation of confidence intervals (95% QBCI). The output of the analysis does not have a logical interpretation of parameter estimates when applying binomial distribution as input. The level of significance and the proportion of the association that was mediated can be directly interpreted. The proportion the association that is mediated is the division of 2 parameter estimates and directly presents the strength of the mediation (proportion of the association that is mediated).

## Results

**Figure 2.**
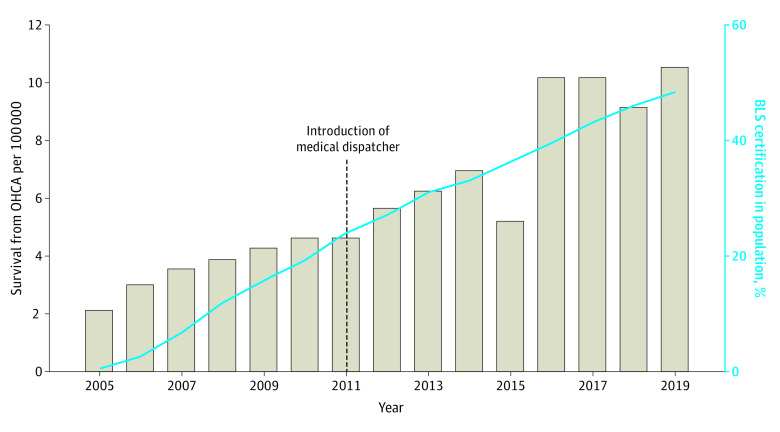
Distribution of Basic Life Support (BLS) Certificates in the Danish Population and Survival From Out-of-Hospital Cardiac Arrest (OHCA), From 2005 to 2019 In 2011 the Danish emergency medical services dispatch system was transformed to include health care educated personnel as dispatchers with standard protocols for telephone-assisted cardiopulmonary resuscitations guidance.

A total of 57 456 OHCAs in between 2005 and 2019 in Denmark were included, resulting in 51 057 OHCAs after excluding OHCAs witnessed by EMS. There were 3 672 004 BLS certificates between 2005 and 2019, resulting in 2 717 933 included certificates as the overall sample size in the study after verification and extrapolation from years where verification was not possible (Table, Figure 2). BLS certificates in the population increased from 35 000 in 2005 to 239 160 in 2019, and survival from OHCAs increased from 2.1% per 100 000 inhabitants in 2005 to 10.6% per 100 000 inhabitants.

**Table.** Out-of-Hospital Cardiac Arrest (OHCA) and Basic Life Support Course (BLS) Participants in Denmark, From 2005 to 2019

Year	OHCA					BLS course certificates		
	Total, No.	Non-EMS-witnessed, No.	30-d survival, %	Survival per 100 000 inhabitants	Bystander CPR, %	Certificates, No.^a^	Certificate holders, No.^a^	
							Verified	Extrapolated
2005	3034	2744	4.5	2.1	27.0	35 000	NA	25 946
2006	2751	2476	6.6	3.0	29.1	165 500	NA	118 983
2007	2874	2568	7.6	3.6	32.1	309 000	NA	229 070
2008	2882	2543	8.4	3.9	36.5	382 500	NA	283 557
2009	3180	2785	8.5	4.3	38.7	290 500	NA	215 355
2010	3390	2924	8.8	4.6	43.6	255 000	NA	189 038
2011	3329	2948	8.8	4.6	57.9	352 450	NA	261 280
2012	3734	3297	9.6	5.7	64.0	244 000	NA	180 884
2013	3923	3464	10.2	6.3	65.7	296 300	NA	219 655
2014	4033	3563	11.0	7.0	65.9	141 300	NA	104 749
2015	3647	3196	9.3	5.2	70.0	249 250	NA	184 776
2016	5099	4547	13.5	10.2	78.2	226 715	197 127	NA
2017	5345	4811	12.8	10.2	78.0	250 849	203 261	NA
2018	5230	4723	11.7	9.2	78.8	234 480	159 971	NA
2019	5005	4468	14.4	10.6	80.0	239 160	144 281	NA

Abbreviations: CPR, cardiopulmonary resuscitation; EMS, emergency medical services; NA, not applicable.

^a^ Number of certificates that have been registered since 2005. Verification of individuals was not possible prior to 2016.

### Analysis of Associations

**Figure 3.**
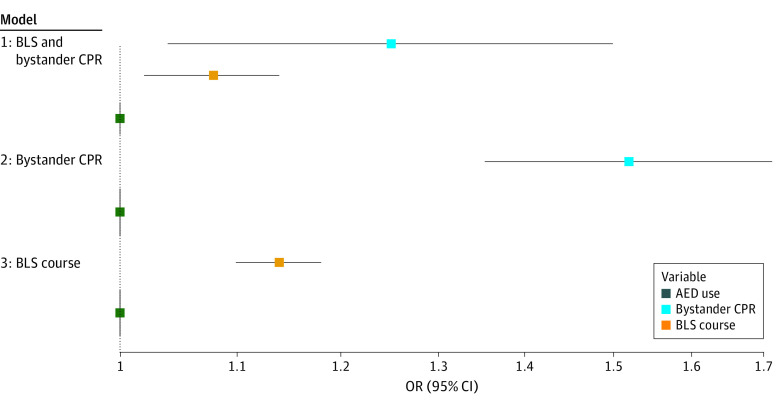
Forest Plot of Models Presenting Association of Training With 30-day Survival From Out-of-Hospital Cardiac Arrest This forest plot presents several models including and excluding variables from mediation analysis. AED indicates automatic external defibrillator; BLS, basic life support; CPR, cardiopulmonary resuscitation; OR, odds ratio.

Model 1, which included both BLS course participation rate and bystander CPR rate, showed that a 5% increase of BLS course participating rate was associated with a significant increase of 30-day survival rate, with an OR of 1.08 (95% CI, 1.02-1.14; *P* = .007), and a 5% increase in bystander CPR rate resulted in an OR of 1.25 (95% CI, 1.04-1.50; *P* = .02) (Figure 3). Model 2, which only included bystander CPR rate as the exposure, showed that a 5% increasing in bystander CPR rate was positively associated with 30-day survival with an adjusted OR of 1.52 (95% CI, 1.35-1.71; *P* < .001). Model 3, which only included BLS course participation rate as exposure, showed that a 5% increase in BLS course participation rate was positively associated with 30-day survival with an adjusted OR of 1.14 (95% CI, 1.10-1.18; *P* < .001). In the combined model, the effect size of the association between both BLS course participation rate and bystander CPR rate and survival decreased, indicating a possible mediation that would help determine which model to use. Sensitivity analysis showed that models using the using the extrapolated values were very robust, with a robustness value for bringing the point estimate of survival to zero at 41.9% and a robustness value for testing the null hypothesis with a 0% coefficient and a partial *R^2^* was 23.2% (eTable in Supplement 1).

### Mediation Analysis

**Figure 4.**
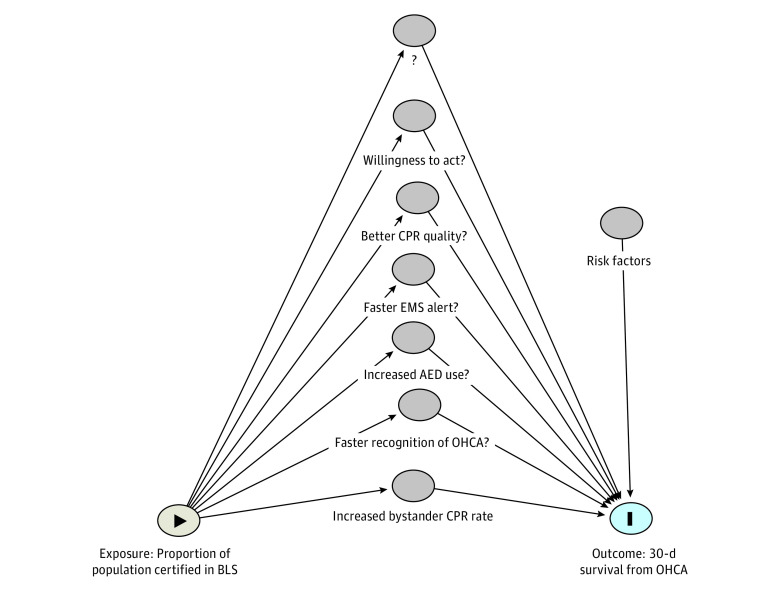
Potential Mediators of the Association Between OHCA Survival and BLS Certification AED indicates automatic external defibrillator; BLS, basic life support; CPR, cardiopulmonary resuscitation; EMS, emergency medical services; OHCA, out-of hospital cardiac arrest.

The mediation analysis showed that the association between BLS course participation and 30-day survival rate was significantly mediated by bystander CPR rate (Figure 4). The parameter estimates were all significant with an average mediation effect of 1.008 (95% QBCI, 1.001-1.020; *P* = .03), average direct effect of 1.012 (95% QBCI, 1.003-1.024; *P* = .005), total effect of 1.010 (95% QBCI, 1.005-1.020; *P* > .001), and an average mediated proportion of 0.39 (95% QBCI, 0.049-0.818; *P* = .01). In other words, the last result indicated that 39% of the association between mass educating laypersons in BLS and survival was mediated through an increased bystander CPR rate. The results indicated that model 3, which only included BLS course participation rate as the exposure, should be applied. Analyses showed that a 5% increase in BLS course participation rate was positively associated with 30-day survival with an adjusted OR of 1.14 (95% CI, 1.10-1.18; *P* < .001). The remaining 61% of the association between mass educating laypersons in BLS and survival resides in other mediators.

## Discussion

This study found a positive association between annual BLS course participation rate and 30-day survival from OHCA in the general population. It was seen that 39% of the association between BLS course participation rate and survival was mediated by increased bystander CPR rate.

This study contributes to the understanding of some of the knowledge gaps that still exist concerning legal regulations facilitate BLS deployment of the general population and the need to isolate the specific interventions for evaluations of isolated association.^22^ The Danish setting in the 10 years after implementation of legally mandated BLS course participation enabled our analysis for some indication of whether the legal regulations factor into survival. The increased 30-day survival rates were significantly associated with the corresponding rise in proportion of the population attending BLS courses. As Sapigliati et al^22^ point out, the Danish setting can best be characterized as a “bundle of interventions” consisting of many interventions, including legal regulation, mass media campaigns, and updated EMS dispatch protocols. The results of this study should be perceived as analysis of one initiative in a setting of multiple initiatives to improve survival from OHCA as well as BLS knowledge and skills in laypersons. In the years from 2006 to 2009, mandated BLS courses were implemented and there was a rise in both proportion of the population educated in BLS and in 30-day survival. This was to be expected due the increasing level of implementation and increasing focus on OHCA in other initiatives. It is known that dispatcher-assisted CPR affect survival.^30^ In the current study period, a national protocol for introducing standardized dispatch CPR was introduced in 2011. There was no major change in the trend for 30-survival in 2011 and beyond (Figure 2).

Mass education in BLS is part of a range of initiatives in Denmark during the study period, and trends of increasing survival were seen before full implementation of mandated BLS courses. The present study shows a clear significant association between mass education in BLS and survival. Furthermore, this study shows that bystander CPR has acted as a mediator in the association between BLS mass education and survival, and accounts for approximately 39% of the association. These results support using bystander CPR rates as an indicator when assessing how BLS mass education factors into patient survival, as suggested by Scapigliati et al.^22^ It also indicates that the association between BLS education and 30-day survival was partly mediated by factors not directly linked to bystander CPR provision.

Bystander CPR was a significant mediator in the association between BLS course participation and 30-day survival rate from OHCA compared with other potential mediators (Figure 4). BLS course content includes several elements known to improve survival, such as recognition, early call for EMS, improved CPR quality, better communication with the EMS dispatch center, and increased AED usage.^21,24,31,32^ One element worth emphasizing is that participants in a BLS course are expected to recognize OHCA sooner and alert EMS faster. The importance of early recognition as a result of BLS education and training have been stressed by many resuscitation researchers^33,34,35,36,37,38^ and several key publications suggest this to affect survival.^1,3,4^ This might explain some of the remaining residual variance in the models. The other elements cannot be measured as easily, and would require granulated and detailed data about bystanders individual training status coupled with the relevant OHCA. Estimating the relative impact of BLS mass education on survival is hence currently limited by available data sources. Several studies indicate that mass education in BLS increases the general willingness to act, which leads to even more unknown factors^24,39,40,41^ and might also affect emergencies beside OHCA.

A 2022 study^24^ argued that a community where BLS training is common there might be a general increased awareness of recognition and acting, even among those whom have not participated in BLS training. This leads to a broader debate on how to overcome barriers for laypersons to act at OHCA and potentially perform bystander CPR. There is evidence suggesting that BLS courses generally do not mirror reality and do not address some of the known barriers for laypersons.^42^ There is similarly evidence suggesting that BLS courses generally increase the willingness to act under all circumstances.^24,43^ BLS mass education might lower barriers to act in a community.

Studies indicate that barriers are context specific and that BLS courses should be modeled around contextual psychosocial barriers as much as technical skill.^44,45^ One seemingly intuitive barrier to address in future courses could be to consider the likelihood that bystander and victim know each other (ie, the bystander is likely to be a family member, friend, or spouse). As 61% of the association between BLS mass education and survival is not based on CPR skills, there is an opportunity for debate on what is expected of participants after a retention period.

### Limitations

This study had several limitations. Because there are some areas with higher or lower rates of BLS education in Denmark, there was geographical variation in instruction that was not considered. Course content partly changed from 2005 to 2019 to adjust for changes in guidelines on resuscitation, which was not addressed in the current study. The European BLS courses teach laypersons to provide mouth-to-mouth ventilation in between chest compressions. This is not consistent with lay CPR education in North America, which limits the comparability of results and conclusions. Our study does not report on any potential change in quality of bystander CPR. This study is also limited in its analysis by the lack of data concerning individuals performing bystander’s CPR at OHCA incidents. There was also a lack of information about whether telephone-assisted CPR was initiated at OHCA incidents. Conclusions were limited to a national context of mass education mandated by law (to avoid an ecological fallacy).

## Conclusions

This cohort study showed a positive association between annual rate of mass education in BLS and 30-day survival from OHCA in the general population, following introduction and implementation of legally mandated BLS course for receiving a driver’s license and for vocational education programs. Approximately 40% of the association between BLS course participation rate and 30-day survival was mediated by the increased rates of bystander CPR at OHCA. Therefore, approximately 60% of the association between BLS course participation rate and 30-day survival was based on factors other than CPR rates.

## Notes

Supplement 1. **eTable.** Sensitivity Analysis Parameters

Supplement 2.
Data Sharing Statement
