# Supplementary material for: Training in Basic Life Support and Bystander-Performed Cardiopulmonary Resuscitation and Survival in Out-of-Hospital Cardiac Arrests in Denmark, 2005 to 2019
Source: JAMA Netw Open. 2023 Mar 16;6(3):e233338. doi: 10.1001/jamanetworkopen.2023.3338 (PMC10020888; doi:10.1001/jamanetworkopen.2023.3338)
Supplement: Supplement 1. — eTable. Sensitivity Analysis Parameters [file jamanetwopen-e233338-s001.pdf]

Supplemental Online Content

Jensen TW, Ersbøll AK, Folke F, et al. Training in basic life support and bystander-performed cardiopulmonary resuscitation and survival in out-of-hospital cardiac arrests in Denmark, 2005 to 2019. *JAMA Netw Open.* 2023;6(3):e233338. doi:10.1001/jamanetworkopen.2023.3338

eTable. Sensitivity Analysis Parameters

This supplemental material has been provided by the authors to give readers additional information about their work.

© 2023 Jensen TW et al. *JAMA Network Open.*

eTable. Sensitivity Analysis Parameters

Sensitivity Analysis Parameters

**Treatment**	**Outcome: Thrity survival from OHCA**					
	**Est.**	**S.E.**	**t-value**	$R^2_{Y\sim D\|X}$	$RV_{q=1}$	$RV_{q=1, \alpha=0.05}$
BLS courses	0.008	0.005	1.651	23.2%	41.9%	0%

Note: df = 9; Bound (1x initial rhythm = shockable): $R^2_{Y\sim Z|X,D}$ = 0.2%, $R^2_{D\sim Z|X}$ = 1.2%

BLS: Basic Life Support

OHCA: Out-of hospital cardiac arrest

© 2023 Jensen TW et al. *JAMA Network Open.*
