# Supplementary material for: Training in Basic Life Support and Bystander-Performed Cardiopulmonary Resuscitation and Survival in Out-of-Hospital Cardiac Arrests in Denmark, 2005 to 2019
Source: JAMA Netw Open. 2023 Mar 16;6(3):e233338. doi: 10.1001/jamanetworkopen.2023.3338 (PMC10020888; doi:10.1001/jamanetworkopen.2023.3338)
Supplement: Supplement 2. — Data Sharing Statement [file jamanetwopen-e233338-s002.pdf]

# Data Sharing Statement

Jensen TW, Ersbøll AK, Folke F, et al. Training in Basic Life Support and Bystande-Performed Cardiopulmonary Resuscitation and Survival in Out-of-Hospital Cardiac Arrests in Denmark, 2005 to 2019. *JAMA Netw Open*. Published March 16, 2023. doi:10.1001/jamanetworkopen.2023.3338

**Data**
**Data available:** Yes
**Data types:** Other (please specify)
**Additional Information:** Meta-data upon reasonable request
**How to access data:** Meta-data upon reasonable request
**When available:** With publication
**Supporting Documents**
**Document types:** None

**Additional Information**
**Who can access the data:** Meta-data upon reasonable request
**Types of analyses:** Meta-data upon reasonable request
**Mechanisms of data availability:** By individual agreement depending on request
